# Aerobic capacity and cardiopulmonary variables are not different between premenopausal, late premenopausal, perimenopausal, and postmenopausal women

**DOI:** 10.14814/phy2.70503

**Published:** 2025-08-18

**Authors:** Catherine A. Rattley, Paul Ansdell, Matthew Armstrong, Malika Felton, Susan Dewhurst, Karen Yendole, Rebecca A. Neal

**Affiliations:** ^1^ Department of Rehabilitation and Sport Sciences, Faculty of Health and Social Science Bournemouth University Bournemouth UK; ^2^ Department of Sport, Exercise and Rehabilitation, Faculty of Health and Life Sciences Northumbria University Newcastle‐upon‐Tyne UK; ^3^ Department of Sport and Exercise Sciences, Faculty of Social Sciences and Health Durham University Durham UK; ^4^ Department of Life and Environmental Sciences, Faculty of Science and Technology Bournemouth University Bournemouth UK

**Keywords:** exercise physiology, menopause, women's health

## Abstract

Menopause may contribute to declining aerobic capacity alongside aging; whether this is related to declines in physical activity or alterations in physiology is unclear. This study examined the effect of menopause on maximal and submaximal cardiopulmonary variables in an incremental aerobic capacity assessment in active women. Sixty‐nine women, aged between 18 and 60 years, categorized as premenopausal (PRE), late premenopausal (LPRE), perimenopausal (PERI), and postmenopausal (POST) completed a cycle ergometer ramp aerobic capacity test, body composition analysis, and blood hormone testing. Naturally menstruating women were tested in the early follicular phase of the menstrual cycle. One‐way ANOVAs were utilized to analyze the effect of menopause phase on outcome variables. Participant groups had similar V̇O_2peak_, physical activity levels, and endogenous sex hormone profiles (*p* > 0.05), but POST had lower muscle mass than PRE, LPRE, and PERI (*p* < 0.05). There were no differences in maximal or submaximal cardiopulmonary variables (*p* > 0.05). Age and V̇O_2peak_ were not correlated (*r* = −0.23, *p* = 0.06). Contrary to prior reports, maintenance of aerobic capacity is possible throughout midlife and menopause in women with high activity levels. Compared to premenopausal and late premenopausal women, perimenopausal and postmenopausal women demonstrated minimal changes in maximal and submaximal cardiopulmonary variables.

## 1 INTRODUCTION

As research on female exercise performance increases, there remains a gap in data availability for women in midlife (McNulty et al., 2024). There is a particular dearth of research utilizing maximal exercise assessment instead of predictive assessments in women in midlife (Aragão et al., 2011; Bondarev et al., 2018). Increasing the availability of high‐quality data in women, inclusive of aerobic capacity assessment, to study performance determinants in women in perimenopause and postmenopause is essential to address inequalities in sport and exercise science research (McNulty et al., 2024) and to encourage maintenance of physical activity through menopause. The maintenance of aerobic capacity throughout midlife has been associated with lower risk of premature mortality, cardiovascular disease events (Gabriel et al., 2023), hypertension, and diabetes (Lee et al., 2021). Higher aerobic capacity in midlife is also associated with better emotional, occupational, and overall quality of life (Flesaker et al., 2021) and higher physical activity levels slow declines in muscle function (Bondarev et al., 2018) and improve cardiometabolic and physical health in women in midlife (Hulteen et al., 2023). It is difficult to delineate the effect of menopause from the effect of age, especially as research in postmenopause centers on samples of women over 60 years old, which likely results in age effects presenting more prominently than menopause effects (Aragão et al., 2015; Lee et al., 2021). Previously, it has been highlighted that trained postmenopausal women have a lower aerobic capacity than trained premenopausal women as a result of age and sex hormone changes; however, the age of the sample was not reported (Rael et al., 2021). More research utilizing a postmenopausal sample under the age of 60 would permit comparisons utilizing a sample of women with chronically low sex hormones, while minimizing the confounding effect of age.

Declines in aerobic capacity are related to increases in body fat mass and loss of skeletal muscle, which can occur with age (Aragão et al., 2011; Zeiher et al., 2019). This highlights menopause as a time of vulnerability due to associated alterations in body composition (Abildgaard et al., 2021; Lovejoy et al., 2008). Indeed, it has been noted that time since menopause is positively associated with declining aerobic capacity (Aragão et al., 2011; Mercuro et al., 2006), suggesting that increasing time spent in deficiency of estrogen and progesterone is likely influential upon the magnitude of decline in aerobic capacity, independent of age. While acute hormone changes such as those throughout the menstrual cycle have not been found to alter maximal oxygen uptake, respiratory exchange ratio, or respiratory frequency (Rael et al., 2021), there is evidence to suggest that aerobic capacity and cardiopulmonary response to exercise are altered in postmenopause (Mercuro et al., 2006; Rael et al., 2021) A comparison of a sample of women throughout the adult lifecycle, inclusive of perimenopause, is yet to be undertaken and debate surrounding the impact of menopause on aerobic capacity continues (Archiza et al., 2021).

There is evidence to suggest a decline in respiratory function in menopause (Triebner et al., 2017) which is likely to have a direct impact on aerobic capacity (Hassel et al., 2015). The decline in respiratory function in midlife appears to be attenuated by losses of fat mass and increases in muscle mass (Park et al., 2021) but, as noted, menopause is often associated with the inverse; although these changes can be mitigated by physical activity (Bondarev et al., 2018; Juppi et al., 2020). Similarly, while declines in aerobic capacity are inevitable, the rate of age‐related decline in aerobic capacity can be slowed by sport and leisure time physical activity (Bahls et al., 2021).

Impairments in aerobic capacity across the menopause are thought to be related to reductions in estrogen that result in impaired mitochondrial function (Abildgaard et al., 2013; Pellegrino et al., 2022), respiration (Monferrer‐Marín et al., 2024), and efficiency (Zhao et al., 2021). This mitochondrial dysfunction is coupled with reductions in stroke volume (McCole et al., 1999) which cannot be compensated for by heart rate due to age‐related reductions in maximal heart rate (Fleg et al., 1995). A reduced oxygen supply contributes to limitations in the aerobic capacity of skeletal muscle (Hawkins & Wiswell, 2003). Additionally, both menopause and aging lead to a progressive decline in skeletal muscle mass, which reduces absolute O_2_ consumption (Hawkins & Wiswell, 2003). Taken together, the reduced skeletal muscle mass coupled with reduced supply of oxygen due to haemodynamic limitations results in an inability to sustain high‐intensity exercise and, therefore, reduced aerobic capacity (Bassett Jr & Howley, 2000; Cicoira et al., 2001; Hawkins et al., 2001).

The primary focus of this work was to compare maximal and submaximal determinants of aerobic capacity between populations of women at different stages of the menopause to evaluate whether menopause acts as an influential variable requiring consideration in aerobic capacity assessment. The secondary aim was to increase the accessibility of exercise response data in perimenopause and postmenopause. This study is the first to employ an active sample of women throughout the adult lifecycle in low endogenous sex hormone states to compare the effects of transiently lower estrogen in the menstrual cycle to chronically low estrogen of postmenopause, inclusive of an irregularly menstruating sample of perimenopausal women. This is important to establish because these differing hormonal profiles present a different overall exposure to estrogen and progesterone across a menstrual cycle.

## 2 METHODS

A convenience sample of 80 female participants (premenopause, late premenopause, perimenopausal, and postmenopausal) volunteered to take part in this study by poster advertising. Participants were required to be healthy as assessed by the criteria from Greig et al. (1994), exclusion criteria included myocardial infarction, cardiac illness, thrombophlebitis, or pulmonary embolus in the last 10 years, any history of cerebrovascular disease, major systemic disease, osteoarthritis, or use of daily medication aside from hormonal therapies. Participants were excluded if they presented hormone levels outside expected ranges for the early follicular phase. Accepted hormone ranges for premenopausal, late premenopausal, and perimenopausal participants were below 100 pg/mL for oestradiol and below 1.4 ng/mL for progesterone (ABCAM, Cambridge, UK) in line with the early follicular phase or “low hormone phase”.

**FIGURE 1:**
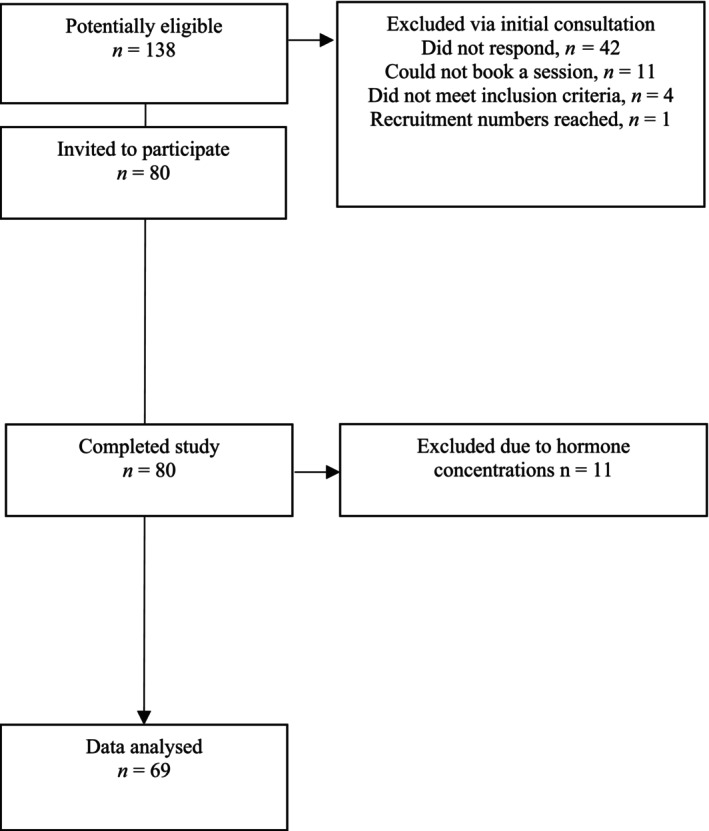
Participant flow throughout the study.

Eleven participants were excluded from analysis due to hormone levels outside of expected ranges; of these, nine were naturally menstruating and two using a hormonal intrauterine system (IUD). Postmenopausal women were not excluded based on hormone levels as there was no possibility of incorrect phase. Participants were not excluded for low oestradiol or progesterone measurements. This resulted in 69 eligible participants (Figure 1). A post hoc power calculation utilizing V̇O_2_ (mL·kg·min^−1^) at first ventilatory threshold provided an effect size (*f* = 0.93). With an alpha level of 0.05, the study was determined to be sufficiently powered to 0.99.

Participants were defined as “active” by self‐report, and where possible, this was subsequently confirmed by physical activity diary with a minimum of 480 leisure time metabolic equivalents (MET). Leisure time MET minutes per week were calculated from diaries; moderate activity level was considered to be 480–720 MET minutes per week, while over 720 MET minutes per week was high (Huxley, 2015).

Menopause status of each individual was defined as (Ambikairajah et al., 2022; Harlow et al., 2012; National Institute for Health and Care Excellence (NICE), 2015):

1. Premenopausal (PRE): between the ages of 18 and 34 years
2. Late premenopausal (LPRE): aged between 35 and 45 years with menstrual regularity or not symptomatic if using hormonal contraceptive
3. Perimenopause (PERI): aged between 40 and 60 years with persistent >7 days difference in length of consecutive cycles or interval of amenorrhea of >60 days and/or vasomotor, musculoskeletal, or mood symptoms (NICE, 2019) if using hormonal contraceptive
4. Postmenopause (POST): aged between 45 and 60 years after 12 consecutive months of amenorrhea

Written informed consent was taken before experimentation. Ethical approval and study standards conformed to the seventh revision of the declaration of Helsinki and were approved by Bournemouth University (19/06/2023). Participants could withdraw at any time. All participants were allocated a code to ensure anonymity; all data were collected under this code. Naturally menstruating women completed the experimental session within 7 days of starting a menstrual bleed to more closely align with the low oestradiol state in postmenopause. Those who were postmenopausal, using hormonal contraceptives, or had not had a bleed for over 60 days were tested at their earliest convenience. Participants were requested to refrain from consuming caffeine and food at least 2 h before each experimental session, and refrain from exercising in the 48 h before maximal exercise testing.

All included participants on PRE, LPRE, and PERI evidenced endogenous hormone levels indicative of the early follicular phase; hormonal therapy users were all included under their relevant menstrual categories. More research is required in hormonal contraceptive users to evaluate their effects on exercise performance (Flood et al., 2024); however, this was not the aim of the present study, which sought to evaluate a representative sample of the population. Detailed participant characteristics based on exogenous hormone use are included in Tables S1 and S2.

### 2.1 Initial measurements

Prior to attending the laboratory, participants completed a health and demographic questionnaire to ensure their eligibility. On attendance at the laboratory initial assessment, a blood pressure measurement (OMRON M2+, Omron Corporation, Kyoto, Japan) was conducted to ensure participants presented as normotensive. Participants then had measurements of anthropometrics taken by stadiometer (217, SECA, Hamburg, Germany) and body mass scales (803, SECA, Hamburg, Germany) followed by body composition measurement using bioelectrical impedance scales (InBody770, InBody Ltd., Seoul, South Korea).

### 2.2 Plasma measurement

All participants were tested for oestradiol and progesterone levels by venepuncture blood sample taken by a trained phlebotomist before performing the maximal exercise test. Blood samples were immediately centrifuged at 1500 rpm for 10 min at 4°C, after which 1.5 mL plasma samples were removed and stored in Eppendorff tubes at −80°C on the day of testing. Subsequently, plasma samples were tested in duplicate by colorimetric enzyme‐linked immunosorbent assay (ELISA) (ab108667, Human Estradiol ELISA Kit, ABCAM, Cambridge, UK; ab108670, Human Progesterone ELISA Kit, ABCAM, Cambridge, UK) and hormone concentration was determined via plate reader (ELX800 Microplate reader, BioTek, Vermont, United States). Minimum detectable plasma concentrations were 8.68 pg mL^−1^ for oestradiol and 0.05 ng mL^−1^ for progesterone. Intra‐assay coefficient of variation was 23.7% for oestradiol and 14.9% for progesterone.

### 2.3 Maximal exercise test

Participants completed an aerobic capacity (V̇O_2peak_) test on a cycle ergometer (Pollock et al., 2015). The test began with a 3‐min period of cycling increasing up to 50 W for the warm‐up, after which power output continually increased; participants cycled at a self‐selected cadence over 70 rpm. Pollock et al. (2015) suggested a rate of increase of 1–2 Watts every 3–5 s dependent on training status. Due to the involvement of nonelite populations and evidence from pilot testing, this was adapted to be 1 Watts every 5–7 s dependent on self‐reported activity level (7 s for moderate, 5 s for high). Participants were instructed to indicate when they thought they were approximately 30 s from exhaustion. At this point, verbal encouragement increased. This continued until the participant could no longer continue despite strong verbal encouragement. The protocol duration was between 12 and 14 min long, with no significant differences in test duration between groups (*p* = 0.08). Expired gases and heart rate were measured continuously throughout using a metabolic cart (K5, Cosmed, Firenze, Italy) and chest heart rate monitor (H10, Polar, Kempele, Finland). A fingertip lactate measurement was taken immediately after the test (Biosen C‐Line, EKF diagnostics, Barleben, Germany). A rating of perceived exertion was taken every 2 min during the test. Peak power output was recorded following the test (Lode Ergometry Manager, LEM10, Groningen, The Netherlands). Attainment of primary and secondary criteria is available in Table S3. Following this, each participant also completed a three‐week activity diary to assess activity level. Twelve participants (2 PRE, 5 LPRE, 3 PERI, and 2 POST) failed to provide activity diaries.

### 2.4 Data management

All expired gas and heart rate data were averaged to 15‐s intervals for export and for analysis of HR‐V̇O_2_ data. Data were averaged to 5‐min time windows for analysis of cardiopulmonary variables including respiratory quotient (RQ), respiratory frequency (RF), tidal volume (TV), minute ventilation (V̇E) and ventilatory equivalent for carbon dioxide (V̇E/V̇CO_2_) (COSMED Omnia, Version 2.2, Cosmed, Firenze, Italy). V̇O_2_ (mL·min^−1^) was plotted against CO_2_ (mL·min^−1^) and V̇E/V̇CO_2_ against V̇E/V̇O_2_ for identification of ventilatory thresholds (VT1 and VT2); this was completed using automatic detection by COSMED Omnia software, then manually verified by two researchers (CR and MA). VT1 represents the exercise intensity at which ventilation begins to increase disproportionately to oxygen uptake, while VT2 marks the intensity at which a further disproportionate rise in ventilation occurs, corresponding to a shift toward predominantly anaerobic metabolism.

Predicted V̇O_2peak_ was calculated using the FRIEND equation (Myers et al., 2017).

### 2.5 Statistical analysis

All data presented are mean and standard deviation (SD). Data were analyzed for normality using Shapiro–Wilk testing. A one‐way analysis of variance (ANOVA) was employed for participant characteristics and maximum criterion variables with Tukey's multiple comparisons test. Where there was unequal variance as identified by Bartlett's test, a Welch's ANOVA was employed with Dunnett's T3 multiple comparisons test. One‐way ANOVAs were utilized to analyze each ventilatory threshold (VT1 and VT2) and maximum for menopause group differences (PRE, LPRE, PERI, and POST) for variables presented in Table 2. Where data were non‐normally distributed, the Kruskal–Wallis test was employed. Pearson's correlation coefficient (*r*) was utilized for the relationship between variables (V̇O_2_, age) for the whole cohort and for defined groups. An *r* ≥ 0.4 was considered moderate, and *r* ≥ 0.7 was considered strong. Simple linear regression between variables was performed to assess the strength of the relationship (GraphPad Prism version 9.0.0 for MacOS, San Diego, California, USA). Individual HR and V̇O_2_ data throughout the test were collated to assess the relationship between HR and V̇O_2_, for which Pearson's correlation coefficient (*r*) was utilized.

In the PRE group, combined oral contraceptive users were retrospectively matched for age to participants from the naturally menstruating sample, and in the POST group, users of hormone therapy matched to those who did not use hormone therapy for subgroup analysis. Unpaired T‐tests were utilized to analyze values at each ventilatory threshold (VT1 and VT2) and maximum, and exogenous hormone differences (exogenous hormone user, non‐user) for key aerobic capacity and ventilatory variables (Table 3).

## 3 RESULTS

### 3.1 Participant characteristics

Age was significantly different between all groups (*p* < 0.05, Table 1). Body fat mass was higher in LPRE than PRE (*p* < 0.001) but there were no differences between the other groups. Muscle mass was lower in the POST group than in all other groups (*p* < 0.05); fat free mass was lower in POST than in LPRE (*p* = 0.02) and PERI (*p* = 0.02) groups. There were no differences in hormone measurements and V̇O_2peak_.

**TABLE 1:** Participant characteristics based on menopause status.

	PRE (*n* = 18)	LPRE (*n* = 16)	PERI (*n* = 14)	POST (*n* = 21)	*p*
Age (years)	27.3 ± 4.0^†,‡,§^	40.2 ± 3.6*^,‡,§^	47.4 ± 4.3*^,†,§^	55.0 ± 3.2*^,†,‡^	**<0.001**
Height (cm)	170.2 ± 5.2^§^	167.9 ± 6.5	169.8 ± 5.7^§^	164.9 ± 3.9*^,‡^	**0.013**
Weight (kg)	67.0 ± 6.5	70.9 ± 11.7	72.0 ± 15.2	68.3 ± 10.0	0.540
BMI (kg•m^−2^)	23.2 ± 2.1	25.2 ± 4.0	19.9 ± 8.0	25.2 ± 4.7	0.315
Body fat (%)	24.1 ± 7.7	26.7 ± 8.7	26.8 ± 9.2	30.2 ± 10.4	0.180
Body fat mass (kg)	16.5 ± 6.5	25.3 ± 4.1*	19.9 ± 11.5	21.6 ± 9.8	**0.013**
Muscle mass (kg)	28.1 ± 2.5^§^	28.6 ± 3.0^§^	28.8 ± 3.8^§^	25.6 ± 2.1*^,†,‡^	**0.002**
Fat free mass (kg)	50.5 ± 4.2	51.5 ± 5.1^§^	51.7 ± 6.5^§^	44.3 ± 10.7*^,†,‡^	**0.006**
Oestradiol (pg/mL)	26.3 ± 28.3	14.6 ± 7.4	24.7 ± 20.5	17.6 ± 21.6	0.326
Progesterone (ng/mL)	0.7 ± 0.4	0.4 ± 0.4	0.3 ± 0.3	1.0 ± 2.2	0.348
Menstrual cycle day	3 ± 1^†^	5 ± 2*	5 ± 1	NA	**0.022**
Metabolic equivalent minutes per week	2735 ± 1030	2354 ± 1230	1905 ± 792	2063 ± 1055	0.249

*Note*: *p* value indicates one way ANOVA significance, symbols indicate significance of multiple comparisons * difference to PRE, † difference to LPRE, and ‡ difference to PERI, § difference to POST. Bold values indicate significance at *p* < 0.05.

Abbreviations: BMI, body mass index; LPRE, late premenopause; PERI, perimenopause; POST, postmenopause; PRE, premenopause.

### 3.2 Cardiopulmonary variables

Cardiopulmonary variables, RF, TV, V̇E, and RQ were the same for all groups at both ventilatory thresholds and maximum (Table 2). HR was higher in PRE than in POST at VT1 (*p* = 0.005) and VT2 (*p* < 0.001). HR_max_ was also higher in the PRE group compared to LPRE (*p* = 0.02), PERI (*p* = 0.01), and POST (*p* < 0.001) groups. End test lactate was higher in the PRE group than the POST group (*p* = 0.01) and peak power was higher in LPRE than in POST (*p* = 0.02).

**TABLE 2:** Variables at ventilatory thresholds (VT1 and VT2) and at maximum for each menopause status.

	PRE	LPRE	PERI	POST	*p*
VT1					
V̇O_2_ (L·min^−1^)	1.42 ± 0.26	1.44 ± 0.39	1.38 ± 0.36	1.29 ± 0.28	0.481
V̇O_2_ (mL·kg^−1^·min^−1^)	21.5 ± 4.8	20.8 ± 6.1	19.7 ± 6.3	19.2 ± 4.3	0.558
Percentage of V̇O_2peak_ (%)	55 ± 9	54 ± 12	55 ± 12	55 ± 8	0.986
Percentage of predicted V̇O_2peak_ (%)	58 ± 12^§^	64 ± 21	71 ± 20	75 ± 15*	**0.02**
HR (b·min^−1^)	123 ± 18^§^	110 ± 19	112 ± 19	103 ± 16*	**0.010**
Percentage of HR_max_ (%)	67 ± 9	63 ± 10	65 ± 10	62 ± 9	0.479
RQ	0.88 ± 0.08	0.87 ± 0.09	0.86 ± 0.10	0.84 ± 0.08	0.221
RF (1/min)	26.0 ± 3.1	23.1 ± 4.8	22.7 ± 5.7	22.7 ± 5.4	0.127
TV (L)	1.4 ± 0.3	1.6 ± 0.3	1.5 ± 0.3	1.4 ± 0.3	0.239
V̇E (L·min^−1^)	36.1 ± 8.1	35.2 ± 7.7	33.8 ± 11.9	31.2 ± 8.7	0.108
V̇E/V̇CO_2_	27.4 ± 1.9	27.8 ± 2.6	26.9 ± 2.6	27.2 ± 2.5	0.777
Power (W)	80 ± 23	79 ± 30	77 ± 30	68 ± 18	0.453
Relative power (W·kg)	1.2 ± 0.4	1.1 ± 0.4	1.1 ± 0.5	1.0 ± 0.3	0.459
VT2					
V̇O_2_ (L·min^−1^)	2.20 ± 0.37	2.23 ± 0.49	2.11 ± 0.44	1.94 ± 0.39	0.153
V̇O_2_ (mL·kg^−1^·min^−1^)	33.1 ± 5.6	32.3 ± 9.1	29.6 ± 6.0	29.1 ± 6.9	0.252
Percentage of V̇O_2peak_ (%)	85 ± 5	83 ± 9	83 ± 9	82 ± 10	0.750
Percentage of predicted V̇O_2peak_ (%)	90 ± 14^§^	99 ± 31	108 ± 22	113 ± 22*	**0.01**
HR (b·min^−1^)	164 ± 15^§^	150 ± 18	151 ± 17	140 ± 16*	**<0.001**
Percentage of HR_max_ (%)	89 ± 7	87 ± 7	88 ± 8	85 ± 8	0.243
RQ	1.04 ± 0.03	1.02 ± 0.04	1.03 ± 0.04	1.01 ± 0.05	0.302
RF (1/min)	32.8 ± 5.2	30.0 ± 6.8	31.0 ± 6.0	31.3 ± 6.3	0.593
TV (L)	2.0 ± 0.3	2.1 ± 0.4	2.0 ± 0.4	1.9 ± 0.3	0.232
V̇E (L·min^−1^)	66.5 ± 14.1	63.1 ± 13.0	62.1 ± 16.6	58.2 ± 13.6	0.354
V̇E/V̇CO_2_	27.8 ± 2.6	27.0 ± 1.5	27.4 ± 2.8	28.4 ± 3.4	0.459
Power (W)	159 ± 23	152 ± 33	155 ± 34	146 ± 28	0.597
Relative power (W·kg)	2.4 ± 0.5	2.2 ± 0.6	2.2 ± 0.6	2.2 ± 0.6	0.629
MAX					
V̇O_2_ (L·min^−1^)	2.60 ± 0.38	2.66 ± 0.41	2.52 ± 0.35	2.37 ± 0.39	0.108
V̇O_2_ (mL·kg^−1^·min^−1^)	38.0 ± 6.6	38.4 ± 7.9	35.6 ± 6.0	35.2 ± 6.3	0.224
Predicted V̇O_2peak_ (mL·kg^−1^·min^−1^)	37 ± 2.1^†,‡,§^	30.7 ± 3.0*^,‡,§^	27.6 ± 3.8*^,†^	25.6 ± 2.8*^,†^	**<0.001**
Percentage of predicted V̇O_2peak_ (%)	106 ± 16^†,‡,§^	118 ± 32*	130 ± 16*	137 ± 21*	**<0.001**
HR (b·min^−1^)	184 ± 10^†,‡,§^	172 ± 11*	171 ± 12*	165 ± 13*	**<0.001**
RQ	1.15 ± 0.08	1.16 ± 0.07	1.15 ± 0.08	1.13 ± 0.07	0.823
RF (1/min)	41.6 ± 8.2	44.0 ± 5.1	42.0 ± 8.6	44.2 ± 6.5	0.703
TV (L)	2.2 ± 0.4	2.3 ± 0.4	2.2 ± 0.3	2.1 ± 0.3	0.150
V̇E (L·min^−1^)	94.6 ± 18.9	100.4 ± 14.0	91.9 ± 18.9	89.8 ± 14.8	0.144
V̇E/V̇CO_2_	30.8 ± 3.4	31.5 ± 2.4	30.6 ± 3.1	32.7 ± 3.8	0.230
Power (W)	204 ± 20	212 ± 23^§^	209 ± 28	186 ± 31^†^	**0.012**
Relative power (W·kg)	3.1 ± 0.4	3.1 ± 0.6	3.0 ± 0.6	2.8 ± 0.6	0.324
Lactate (mmol·L^−1^)	9.3 ± 2.2^§^	8.3 ± 2.6	7.3 ± 1.7	7.0 ± 2.2*	**0.009**

*Note*: *p* indicates significance of one‐way ANOVA. Symbols indicate significant differences at *p* < 0.05; * indicates a difference to PRE, † a difference to LPRE, ‡ a difference to PERI, and § a difference to POST. Bold values indicate significance at *p* < 0.05.

Abbreviations: HR, heart rate; LPRE, late premenopause; MAX, maximum; PERI, perimenopause; POST, postmenopause; PRE, premenopause; RF, respiratory frequency; RQ, respiratory quotient; TV, tidal volume; V̇E, minute ventilation; V̇E/V̇CO_2_, ventilatory equivalent for carbon dioxide; VO_2_, volume of oxygen; VT, ventilatory threshold.

V̇O_2peak_ (mL·kg^−1^·min^−1^) was not correlated with age for the whole sample (*r* = −0.23, *R* ^2^ = 0.05, *p* = 0.06) or for any individual group (PRE: *r* = −0.13, *R* ^2^ = 0.02, *p* = 0.62; LPRE: *r* = 0.07, *R* ^2^ < 0.01, *p* = 0.85; PERI: *r* = −0.23, *R* ^2^ = 0.05 *p* = 0.43; POST: *r* = 0.22, *R* ^2^ = 0.05, *p* = 0.33).

HR and V̇O_2_ were moderately positively correlated for the whole sample (*r* = 0.73, *R* ^2^ = 0.54, *p* < 0.001) and all groups throughout the incremental exercise test (PRE: *r* = 0.77, *R* ^2^ = 0.59, *p* < 0.001; LPRE: *r* = 0.69, *R* ^2^ = 0.47, *p* < 0.001; PERI: *r* = 0.76, *R* ^2^ = 0.59, *p* < 0.001; POST: *r* = 0.73, *R* ^2^ = 0.53, *p* < 0.001).

### 3.3 Impact of exogenous hormone use

As the PRE and POST groups contained females using exogenous hormonal contraceptives and therapies, respectively, comparisons were made with age‐matched females without exogenous hormone usage to determine whether these had any influence on the between‐group comparisons above. VT2 occurred at a higher percentage of maximum HR in POST than in POST HRT (*p* = 0.04). There were no other differences in submaximal and maximal cardiopulmonary variables between women who used combined oral contraceptives and those who were naturally menstruating, nor between those who did and did not use hormone therapy (Table 3).

**TABLE 3:** Variables at ventilatory thresholds and at maximum for users of combined oral contraceptives, naturally menstruating, postmenopause hormone therapy (HT) users, and postmenopausal women. *p* indicates significance of unpaired *t*‐test.

	Combined oral contraceptives (*n* = 5)	Naturally menstruating (*n* = 5)	*p*	POST HT (*n* = 10)	POST (*n* = 10)	*p*
VT1						
V̇O_2_ (L·min^−1^)	1.35 ± 0.32	1.39 ± 0.24	0.800	1.25 ± 0.28	1.29 ± 0.27	0.767
V̇O_2_ (mL·kg^−1^·min^−1^)	19.7 ± 6.3	21.0 ± 4.4	0.692	18.6 ± 4.4	19.4 ± 4.2	0.661
Percentage of V̇O_2peak_ (%)	55 ± 8	53 ± 10	0.736	57 ± 10	53 ± 7	0.314
Percentage predicted V̇O_2peak_ (%)	58 ± 12	61 ± 14	0.712	73 ± 15	75 ± 15	0.940
HR (b·min)	118.3 ± 31.5	115.2 ± 13.4	0.825	102.8 ± 18.8	103.9 ± 14.2	0.885
Percentage of HR_max_ (%)	66 ± 14	64 ± 6	0.777	61 ± 11	64 ± 5	0.443
RQ	0.93 ± 0.07	0.87 ± 0.09	0.286	0.85 ± 0.09	0.82 ± 0.06	0.436
RF (1/min^−1^)	25.3 ± 2.9	23.1 ± 4.9	0.361	21.5 ± 6.0	24.2 ± 4.7	0.282
TV (L)	1.4 ± 0.2	1.5 ± 0.2	0.576	1.5 ± 0.3	1.3 ± 0.2	0.115
V̇E (L·min^−1^)	35.4 ± 6.8	33.9 ± 9.2	0.763	31.0 ± 10.2	30.7 ± 7.6	0.684
V̇E/V̇CO_2_	27.3 ± 2.6	27.1 ± 1.1	0.878	26.9 ± 2.0	27.3 ± 3.0	0.730
Power (W)	80 ± 23	77 ± 28	0.881	67 ± 20	70 ± 17	0.741
Relative power (W·kg)	1.1 ± 0.3	1.2 ± 0.4	0.905	1.0 ± 0.3	1.1 ± 0.3	0.681
VT2						
V̇O_2_ (L·min^−1^)	2.0 ± 0.4	2.3 ± 0.4	0.235	1.8 ± 0.4	2.0 ± 0.3	0.168
V̇O_2_ (mL·kg^−1^·min^−1^)	28.7 ± 9.2	34.1 ± 7.4	0.283	26.6 ± 7.0	30.7 ± 6.2	0.186
Percentage of V̇O_2peak_ (%)	80 ± 10	87 ± 7	0.387	80 ± 8	83 ± 11	0.494
Percentage predicted V̇O_2peak_ (%)	85 ± 16	99 ± 19	0.195	105 ± 22	117 ± 18	0.261
HR (b·min^−1^)	157.7 ± 28.3	157.2 ± 17.0	0.971	136.8 ± 17.5	143.8 ± 14.8	0.346
Percentage of HR_max_ (%)	88 ± 9	85 ± 7	0.57	81 ± 7	88 ± 7	**0.038**
RQ	1.03 ± 0.04	1.05 ± 0.02	0.446	1.01 ± 0.06	1.02 ± 0.03	0.726
RF (1/min^−1^)	31.1 ± 4.6	33.4 ± 6.5	0.500	29.2 ± 7.2	33.7 ± 4.7	0.111
TV (L)	1.9 ± 0.4	2.0 ± 0.3	0.462	1.9 ± 0.4	1.8 ± 0.2	0.582
V̇E (L·min^−1^)	58.2 ± 15.3	66.2 ± 10.6	0.317	54.5 ± 15.6	61.0 ± 11.6	0.299
V̇E/V̇CO_2_	27.0 ± 2.5	27.3 ± 2.0	0.839	28.0 ± 3.3	28.5 ± 3.4	0.743
Power (W)	147 ± 23	153 ± 32	0.744	134 ± 34	156 ± 23	0.109
Relative power (W·kg)	2.2 ± 0.7	2.3 ± 0.6	0.668	2.0 ± 0.6	2.4 ± 0.6	0.148
MAX						
V̇O_2_ (L·min^−1^)	2.43 ± 0.31	2.64 ± 0.30	0.253	2.22 ± 0.38	2.44 ± 0.31	0.187
V̇O_2_ (mL·kg^−1^·min^−1^)	35.5 ± 8.7	39.9 ± 6.2	0.329	33.0 ± 7.6	36.5 ± 3.8	0.203
Predicted V̇O_2peak_ (mL·kg^−1^·min^−1^)	33.4 ± 5.9	34.5 ± 2.6	0.670	25.3 ± 3.1	26.1 ± 2.8	0.360
Percentage of predicted V̇O_2peak_ (%)	106 ± 13	116 ± 18	0.284	130 ± 21	138 ± 15	0.335
HR (b·min^−1^)	178.8 ± 14.2	180.0 ± 8.1	0.865	167.6 ± 11.2	162.9 ± 14.6	0.429
RQ	1.17 ± 0.14	1.17 ± 0.07	0.980	1.13 ± 0.05	1.13 ± 0.09	0.999
RF (1/min^−1^)	43.3 ± 7.2	44.0 ± 6.9	0.878	43.4 ± 7.9	44.3 ± 5.6	0.763
TV (L)	2.1 ± 0.2	2.2 ± 0.3	0.391	2.1 ± 0.3	2.0 ± 0.3	0.671
V̇E (L·min^−1^)	90.4 ± 17.7	96.7 ± 8.0	0.443	88.5 ± 18.5	87.6 ± 14.2	0.903
V̇E/V̇CO_2_	31.0 ± 4.1	30.6 ± 3.6	0.874	33.5 ± 3.0	31.9 ± 3.2	0.264
Power (W)	201.3 ± 16.1	206.2 ± 25.4	0.702	180.4 ± 35.6	186.7 ± 23.0	0.644
Relative power (W·kg)	2.9 ± 0.7	3.1 ± 0.6	0.624	2.7 ± 0.8	2.8 ± 0.4	0.739
Lactate (mmol·L^−1^)	9.0 ± 2.5	8.9 ± 1.6	0.771	7.6 ± 2.0	6.4 ± 2.5	0.268

*Note:* Bold values indicate significance at *p* < 0.05.

Abbreviations: HR, heart rate; HT, hormone therapy; MAX, maximum; POST, postmenopause; RF, respiratory frequency; RQ, respiratory quotient; TV, tidal volume; V̇E, minute ventilation; V̇E/V̇CO_2_, ventilatory equivalent for carbon dioxide; V̇O_2_, volume of oxygen; VT, ventilatory threshold.

## 4 DISCUSSION

This study offers insight into cardiorespiratory responses to maximal exercise in physically active females in premenopause, late premenopause, perimenopause, and postmenopause with similar endogenous hormone profiles. Menopause appeared to have no effect on aerobic capacity (V̇O_2peak_) or submaximal ventilatory thresholds in this sample of active females across the adult lifecycle. There were no differences in cardiopulmonary variables within an aerobic capacity test, suggesting minimal impact of age or chronically low hormone levels compared to acutely low hormone levels.

The rate of decline of aerobic capacity in women in mid‐life is debated, but it is reported to range between 0.8% and 1.6% per year (Hawkins & Wiswell, 2003; Loe et al., 2013), regardless of activity level. Notably, in data from Loe et al. (2013) this decline accelerates from a 4% decline in V̇O_2peak_ between age groups 30 and 39 (40.0 mL·kg^−1^·min^−1^) and 40 and 49 (38.4 mL·kg^−1^·min^−1^) to a 10% decline between 40 and 49 and 50 and 59 age groups (34.4 mL·kg^−1^·min^−1^). Hawkins and Wiswell (2003) also suggested that it does not appear possible for women in midlife to slow this accelerated decline in V̇O_2peak_ and that this inability to mitigate loss of aerobic capacity may be related to estrogen losses. In the present study, however, we show no decrease in V̇O_2peak_ between premenopausal and postmenopausal women with comparable physical activity levels, implying that maintenance of aerobic capacity is possible through midlife. This is similar to reports that age‐related declines in aerobic capacity can also be slowed by physical activity (Bahls et al., 2021) which may be linked to the benefits of physical activity on cardiovascular health (Hellsten & Nyberg, 2015; Seals et al., 2019; Craighead et al., 2019).

The age reduction in V̇O_2peak_ relies on two assumptions: declines in lean body mass and declines in maximum heart rate (HR_max_) (Hawkins & Wiswell, 2003) linked to arterial stiffening and endothelial dysfunction (Seals et al., 2019). Declines in lean body mass cause a reduction in oxygen utilization by skeletal muscle and therefore lower V̇O_2_ at an absolute workload (Hawkins & Wiswell, 2003). This reduction in V̇O_2_ in postmenopause with similar absolute workloads was not observed at ventilatory thresholds or maximum despite significantly lower muscle mass. However, maximum lactate was significantly lower in postmenopause compared to premenopause; this aligns with reports of lower lactate accumulation in women in mid‐life due to age and sex (Bovens et al., 1993; Sargent & Scroop, 2007). This lower lactate accumulation may result from muscle mass losses and atrophy of type II muscle fibers (Pogliaghi et al., 2023). Lower lactate accumulation in female participants has also been related to a reduced reliance on carbohydrate metabolism (Carter et al., 2001; Jacobs et al., 1983) rather than a higher rate of clearance (Bovens et al., 1993), but these factors are unlikely to influence maximal lactate.

The effects of declines in HR_max_ on V̇O_2peak_ rely on the linearity of the HR and V̇O_2_ relationship (Vehrs et al., 2022). Variability exists in the HR‐V̇O_2_ relationship, as a product of age, sex, or individual variability (Gastinger et al., 2010), which may result in inaccurate prescription of aerobic exercise (Vehrs et al., 2022). Therefore, it is essential to evaluate the linearity of this relationship throughout the menopausal transition and into postmenopause. Data presented here confirm that the HR and V̇O_2_ relationship remains linear through and after the menopausal transition. While there are sex differences in the linearity of this relationship (Fairbarn et al., 1994), with males evidencing a stronger relationship than females (Andrade et al., 2020; Loe et al., 2013), the current study corroborates that there are no differences in linearity between females under and over 50 years (Fairbarn et al., 1994). Maximum HR of the premenopause group was higher than all other groups, yet the previously reported accompanying difference in V̇O_2peak_ (Mercuro et al., 2006) is not evident in this study. In contrast with previous reports, our sample did not evidence correlations between V̇O_2peak_ and age (Pollock et al., 2015). Therefore, declines in V̇O_2peak_ through midlife up to 60 years old may not be as pronounced in females as previously reported, provided that high physical activity levels are maintained (Rael et al., 2021). In fact, alternative explanations for these accelerated declines should be explored, such as a decline in habitual physical activity on physical activity (Women In Sport, 2018).

In the current work, no differences between menopause groups were evidenced in V̇E, TV, RF, or V̇E/V̇CO_2_. However, it is well reported that aging impacts pulmonary function, and therefore ventilation, by increases in expiratory flow limitation (McClaran et al., 1995, 1998). In addition, menopause has also been linked with declines in pulmonary function whereby forced vital capacity (FVC) reduces by up to −12.5 mL per year through the perimenopausal transition and postmenopause (Campbell et al., 2018; Triebner et al., 2017). Notably, previous evidence suggests a positive correlation between oestradiol and TV across the menstrual cycle, implying that when oestradiol is low in menopause (da Silva et al., 2006), TV could be expected to decline, which, without compensation with increasing respiratory frequency, would result in reduced V̇E. Constrictions on V̇E can result in an inability to ventilate sufficiently in response to stressors and subsequent fatigue (Babb, 2013). Differences in TV and V̇E have not been evidenced with menstrual cycle‐related fluctuations in hormones (da Silva et al., 2006) however, research availability is limited. If V̇E is limited in menopause, in addition to the effects of aging, then it can be expected that ventilatory efficiency may be reduced. Previous research studying menstrual cycle‐related changes in V̇E/V̇O_2_ has, in some cases, lacked adequate control (Dombovy et al., 1987) and was limited by sample size (Beidleman et al., 1999; Dombovy et al., 1987). Evidence exists of a higher V̇E and higher V̇E/V̇CO_2_ in the luteal phase compared to mid‐follicular (Barba‐Moreno et al., 2022; Beidleman et al., 1999), implying a reduced ventilatory efficiency in low hormone phases. This phenomenon may need to be studied with a larger sample; however, in the case of menopause, it is suggested that differences would likely result from age‐related changes (McConnell & Davies, 1992) independent from those exerted by hormones (Davenport et al., 2012).

### 4.1 Experimental considerations

This work sought to investigate the changes in an aerobic capacity test induced by menopause in a representative sample of the female population and therefore increase data availability. Accurate categorization of participants as perimenopausal is difficult due to the lack of available blood hormone concentration normative data. This stems from the unpredictability of sex hormone concentrations in this phase, which is further complicated by significant day‐to‐day hormonal variability that challenges any consistent classification. With increasing focus on the physiological impact of perimenopause, it may be prudent to explore the expected hormonal concentrations associated with this life stage in order to support more precise categorization as early and late perimenopause, currently recommended in the STRAW criteria (Harlow et al., 2012).

Use of age‐stratified analysis, with larger samples than those employed in this study, could also yield additional insights into the effects of chronological aging on aerobic capacity in females through midlife, including how physical activity could mitigate declines. It should be acknowledged that the women in this study may have always engaged in physical activity, such that an analysis of the role of lifelong physical activity and its involvement in mitigating changes in aerobic capacity could be warranted. However, lifelong physical activity data was not collected in this cohort, and this is an area for further research. In the interest of a representative sample, this sample involved hormone therapy and hormonal contraceptive users. Indeed, as noted, this work is limited by its sample size, leading to an inability to consider further subgroup analysis by specific hormone therapy or contraceptive type (Flood et al., 2024), aside from the age‐matched comparisons presented. Further research should focus on the impact of the use of hormonal contraceptive and hormone therapy and of endogenous and exogenous hormone profiles on these variables.

## 5 CONCLUSION

In a physically active sample of female participants, menopause and chronic changes in sex hormones have no effect on maximal oxygen uptake and submaximal ventilatory thresholds measured by incremental exercise testing. This suggests that aerobic capacity changes in menopause may relate to other factors such as reduction of physical activity. Much like conclusions from research in the menstrual cycle, menopause does not appear to influence cardiorespiratory fitness nor the linearity of the HR‐V̇O_2_ relationship. The importance of maintaining or increasing aerobic activity through the menopause transition should be emphasized, and exercise prescription should not be adjusted based on menopausal status but instead individually tailored with consideration for symptom burden.

## AUTHOR CONTRIBUTIONS

CR was involved in conceptualization, methodology, investigation, formal analysis, and writing—original draft preparation. MF, SD, RN, and PA were involved in supervision and writing—review and editing. KY was involved in validation and investigation. MA was involved in writing—reviewing and editing.

## FUNDING INFORMATION

This study was funded by Bournemouth University by a doctoral studentship awarded to the first author.

## CONFLICT OF INTEREST STATEMENT

The authors have no relevant financial or non‐financial interests to disclose.

## ETHICS STATEMENT

This research was approved by Bournemouth University Ethics Committee under ID number 44678 on 29/06/2022. Ethical approval and study standards conformed to the seventh revision of the declaration of Helsinki. Informed consent was obtained from all individual participants included in the study.

## Supporting information


Table S1.

Table S2.

Table S3.

## Data Availability

The datasets generated during and/or analyzed during the current study are available from the corresponding author on reasonable request.
