# Supplementary material for: Aerobic capacity and cardiopulmonary variables are not different between premenopausal, late premenopausal, perimenopausal, and postmenopausal women
Source: Physiol Rep. 2025 Aug 18;13(15):e70503. doi: 10.14814/phy2.70503 (PMC12358808; doi:10.14814/phy2.70503)
Supplement: Supplementary file 1 — Table S1. [file PHY2-13-e70503-s003.docx]

Table S1: Participant characteristics based on contraceptive use, Symbols indicate: * difference to PRE NM, † difference to PRE LARC, ‡ difference to POST HT. 1 perimenopause POP user and 1 perimenopause COC user not included.

|  | **PRE NM (*n* = 20)** | **PRE COC (*n* = 6)** | **PRE LARC (*n* = 4)** | **PRE POP (*n* = 3)** | **PERI NM (*n* = 6)** | **PERI LARC (*n* = 6)** | **POST (*n* = 10)** | **POST HT (*n* = 11)** |
| --- | --- | --- | --- | --- | --- | --- | --- | --- |
| **Age (years)** | 35.2 ± 7.2 | 31.1 ± 9.5 | 30.3 ± 8.3 | 30.3 ± 1.5 | 46.2 ± 4.1 | 49.5 ± 4.1 | 54.7 ± 3.1 | 55.3 ± 3.5 |
| **Height (cm)** | 169.2 ± 6.4 | 167.8 ± 5.3 | 167.1 ± 5.6 | 174.0 ± 2.5 | 170.2 ± 5.4 | 171.4 ± 5.8 | 163.7 ± 4.2 | 166.0 ± 4.1 |
| **Weight (kg)** | 69.9 ± 9.0 | 70.2 ± 12.6 | 63.5 ±7.4 | 64.0 ± 2.7 | 74.7 ± 15.3 | 70.6 ± 12.5 | 67.2 ± 9.6 | 69.4 ± 14.0 |
| **BMI (kg/m^2^)** | 24.5 ± 2.9 | 25.0 ± 4.7 | 22.7 ± 2.0 | 21.2 ± 0.9 | 25.7 ± 4.7 | 24.0 ± 3.6 | 25.1 ± 3.5 | 25.3 ± 5.8 |
| **Body fat (%)** | 26.6 ± 6.8 | 27.6 ± 11.4 | 21.8 ± 7.3 | 16.3 ± 4.0‡ | 28.3 ± 6.1 | 24.6 ± 8.5 | 29.3 ± 9.9 | 31.1 ± 10.4 |
| **Body fat mass (kg)** | 23.1 ± 5.1 | 19.9 ± 9.0 | 15.5 ± 7.4 | 10.5 ± 2.6 | 21.7 ± 8.9 | 18.1 ± 8.7 | 20.5 ± 8.8 | 22.5 ± 12.7 |
| **Muscle mass (kg)** | 28.6 ± 3.2 | 27.7 ± 1.8 | 27.3 ± 1.3 | 29.87 ± 1.8 | 29.2 ± 4.5 | 29.2 ± 3.5 | 25.6 ± 2.0 | 25.6 ± 2.0 |
| **Fat free mass (kg)** | 51.4 ± 5.4 | 49.8 ± 2.9 | 46.5 ± 6.8 | 53.9 ± 2.7 | 52.6 ± 5.9 | 52.7 ± 6.0 | 41.5 ± 14.9* | 46.8 ± 3.4 |
| **V̇O_2peak_ (mL·kg^−1^·min^−1^)** | 38.1 ± 5.6 | 36.2 ± 8.1 | 46.5 ± 6.8 | 39.6 ± 11.7 | 35.3 ± 6.0 | 36.8 ± 5.6 | 36.5 ± 3.8 | 34.0 ± 8.0 |
| **Oestradiol (pg/ml)** | 18.0 ± 16.7 | 7.8 ± 10.8 | 46.7 ± 38.8 | 35.0 ± 14.3 | 23.5 ± 21.0 | 27.5 ± 22.7 | 4.0 ± 2.4‡*†* | 29.9 ± 24.0 |
| **Progesterone (ng/ml)** | 0.6 ± 0.4 | 0.5 ± 0.4 | 0.6 ± 0.4 | 0.7 ± 0.3 | 0.3 ± 0.3 | 0.3 ± 0.2 | 1.0 ± 2.7 | 1.0 ± 1.8 |

^COC combined oral contraceptive, HT hormone therapy, LARC long-acting reversible contraceptive, NM naturally menstruating, PERI perimenopausal, POP progesterone only pill, POST postmenopausal, PRE premenopausal.^
