# Supplementary material for: Aerobic capacity and cardiopulmonary variables are not different between premenopausal, late premenopausal, perimenopausal, and postmenopausal women
Source: Physiol Rep. 2025 Aug 18;13(15):e70503. doi: 10.14814/phy2.70503 (PMC12358808; doi:10.14814/phy2.70503)
Supplement: Supplementary file 2 — Table S2. [file PHY2-13-e70503-s001.docx]

Table S2: Exogenous hormone formulations for contraceptive or hormone therapy users.

|  | **Progestogen only pill formulation (n=4)** | **Monophasic combined pill formulation**  **(n=8)** | **Long-acting reversible contraceptives**  **(n=11)** | **Hormone therapy**  **(n=11)** |
| --- | --- | --- | --- | --- |
| **PRE** | 75μg desogestrel (Cerazette) - 3 users | 35μg ethinylestradiol and 250μg norgestimate (Cilique) (1^st^) - active  30μg ethinylestradiol and 150μg levonorgestrel (Rigevidon) (2^nd^) - active  30μg ethinylestradiol and 75μg  gestodene (Millinette)(3^rd^) - active  30μg ethinylestradiol and 3mg drospirenone (Yacella) (4^th^) – inactive | ~20 µg/day levonorgestrel (Mirena coil) - 2 users  ~30-40 µg/day (Implanon) |  |
| **LPRE** |  | 30μg ethinylestradiol and 150μg desogestrel (Gederal) (3^rd^) - active  30μg ethinylestradiol and 150μg levonorgestrel (Rigevidon) (2^nd^) - active  30μg ethinyl estradiol and 3mg drospirenone (Yasmin) (4^th^) - active | ~20 µg/day levonorgestrel (Mirena coil) |  |
| **PERI** | 75μg desogestrel (Desogestrel) | 35μg ethinylestradiol and 250μg norgestimate (Cilique) (1^st^) - active | ~20 µg/day levonorgestrel (Mirena coil) - 6 users | 1.5mg oestradiol (Oestrogel)  0.75mg oestradiol (Oestrogel)  ~100 µg/day oestradiol (Evorel patch 6.4 mg) |
| **POST** |  |  |  | 3.2mg estradiol hemihydrate, 11.2mg norethisterone acetate (Evorel Conti Patch)  3.2mg estradiol hemihydrate, 11.2mg norethisterone acetate (Evorel Conti Patch)  2mg as estradiol hemihydraten and 1mg norethisterone acetate (Kliofem pill)  2.25mg oestradiol (Oestrogel x3)  100mg micronized progesterone (Utrogestan)  1mg estradiol hemihydrate) and 0.5mg norethisterone acetate (Kliovance pill)  100mg micronized progesterone (Utrogestan) and 0.75mg oestradiol (Oestrogel)  ~20 µg/day levonorgestrel (Mirena coil) – 2 users  2mg estradiol hemihydrate and 1mg norethisterone acetate (Elleste Duet Conti pill)  Patches (undisclosed) |

^LPRE late premenopause, PERI perimenopause, POST postmenopause, PRE premenopause.^
