# Supplementary material for: Aerobic capacity and cardiopulmonary variables are not different between premenopausal, late premenopausal, perimenopausal, and postmenopausal women
Source: Physiol Rep. 2025 Aug 18;13(15):e70503. doi: 10.14814/phy2.70503 (PMC12358808; doi:10.14814/phy2.70503)
Supplement: Supplementary file 3 — Table S3. [file PHY2-13-e70503-s002.docx]

|  | **Early premenopause** | | | | | | | | | | | | | | | | | | |
| --- | --- | --- | --- | --- | --- | --- | --- | --- | --- | --- | --- | --- | --- | --- | --- | --- | --- | --- | --- |
| **Plateau of 150ml·min^−1^ in final 60 seconds** | Y | Y | Y | Y | Y | N | Y | Y | Y | Y | N | Y | N | Y | Y | Y | Y | Y |  |
| **%predicted HRmax** | 91% | 103% | 94% | 95% | 98% | 84% | 103% | 93% | 93% | 98% | 98% | 99% | 96% | 97% | 93% | 98% | 95% | 89% |  |
| **RER** | 1.03 | 1.16 | 1.17 | 1.07 | 1.24 | 1.08 | 1.17 | 1.23 | 1.32 | 1.04 | 1.21 | 1.00 | 1.16 | 1.24 | 1.09 | 1.11 | 1.20 | 1.16 |  |
| **BLa** | 9.9 | 8.01 | 11.5 | 10.76 | 5.87 | 8.62 | 12.45 | 9.02 | 9.8 | 8.67 | 13.71 | 9.67 | 8.06 | 6.9 | 6.05 | 12.08 | 8.15 | 8.99 |  |
| **RPE** | 19 | 19 | 20 | 19 | 20 | 19 | 20 | 17 | 19 | 18 | 18 | 19 | 19 | 19 | 19 | 19 | 19 | 18 |  |
| **Within 10% of predicted HRmax** | Y | Y | Y | Y | Y | N | Y | Y | Y | Y | Y | Y | Y | Y | Y | Y | Y | N |  |
| **RER of 1.1** | Y | Y | Y | Y | Y | Y | Y | Y | Y | Y | Y | N | Y | Y | Y | Y | Y | Y |  |
| **BLa of 8 mmol·L^−1^** | Y | Y | Y | Y | N | Y | Y | Y | Y | Y | Y | Y | Y | N | N | Y | Y | Y |  |
| **RPE of 19** | Y | Y | Y | Y | Y | Y | Y | N | Y | N | N | Y | Y | Y | Y | Y | Y | N |  |

Table S3: End criteria for maximal exercise test to verify attainment of VO_2max_ for each menopause group. Where a plateau of 150ml·min^−1^ was not attained, use of secondary criteria were employed.

|  | **Late premenopause** | | | | | | | | | | | | | | | | |
| --- | --- | --- | --- | --- | --- | --- | --- | --- | --- | --- | --- | --- | --- | --- | --- | --- | --- |
| **Plateau of 150ml·min^−1^ in final 60 seconds** | Y | N | Y | Y | Y | N | Y | Y | Y | Y | Y | Y | N | Y | Y | Y |  |
| **%predicted HRmax** | 85% | 90% | 93% | 98% | 98% | 99% | 86% | 92% | 101% | 95% | 100% | 99% | 97% | 107% | 101% | 92% |  |
| **RER** | 1.08 | 1.42 | 1.11 | 1.18 | 1.10 | 1.15 | 1.28 | 1.08 | 1.20 | 1.26 | 1.08 | 1.14 | 1.12 | 1.16 | 1.14 | 1.12 |  |
| **BLa** | 4.86 | 5.88 | 7.43 | 9.94 | 10.05 | 9.9 | 10.17 | 6.81 | 7.14 | 6.52 | 12.16 | 5.09 | 7.13 | 11.07 | 7.2 | 13.38 |  |
| **RPE** | 16 | 19 | 19 | 18 | 17 | 20 | 20 | 18 | 18 | 17 | 18 | 19 | 20 | 19 | 19 | 20 |  |
| **Within 10% of predicted HRmax** | N | Y | Y | Y | Y | Y | N | Y | Y | Y | Y | Y | Y | Y | Y | Y |  |
| **RER of 1.1** | Y | Y | Y | Y | Y | Y | Y | Y | Y | Y | Y | Y | Y | Y | Y | Y |  |
| **BLa of 8 mmol·L^−1^** | N | N | N | Y | Y | Y | Y | N | N | N | Y | N | N | Y | N | Y |  |
| **RPE of 19** | N | Y | Y | N | N | Y | Y | N | N | N | N | Y | Y | Y | Y | Y |  |

|  | **Perimenopause** | | | | | | | | | | | | | | |
| --- | --- | --- | --- | --- | --- | --- | --- | --- | --- | --- | --- | --- | --- | --- | --- |
| **Plateau of 150ml·min^−1^ in final 60 seconds** | Y | Y | Y | Y | Y | Y | N | N | N | N | Y | Y | Y | Y |  |
| **%predicted HRmax** | 104% | 115% | 101% | 92% | 111% | 94% | 96% | 98% | 97% | 94% | 94% | 91% | 103% | 101% |  |
| **RER** | 1.11 | 1.08 | 1.11 | 1.09 | 1.21 | 1.16 | 1.22 | 1.00 | 1.32 | 1.12 | 1.13 | 1.18 | 1.25 | 1.13 |  |
| **BLa** | 9.1 | 10.17 | 5.65 | 9.11 | 8.43 | 4.94 | 6.46 | 9.4 | 8.29 | 5.84 | 5.23 | 6.44 | 6.88 | x |  |
| **RPE** | 20 | 19 | 19 | 20 | 20 | 19 | 19 | 20 | 20 | 20 | 20 | 19 | 20 | 13 |  |
| **Within 10% of predicted HRmax** | Y | Y | Y | Y | Y | Y | Y | Y | Y | Y | Y | Y | Y | Y |  |
| **RER of 1.1** | Y | Y | Y | Y | Y | Y | Y | N | Y | Y | Y | Y | Y | Y |  |
| **BLa of 8 mmol·L^−1^** | Y | Y | N | Y | Y | N | N | Y | Y | N | N | N | N | Y |  |
| **RPE of 19** | Y | Y | Y | Y | Y | Y | Y | Y | Y | Y | Y | Y | Y | N |  |

|  | **Postmenopause** | | | | | | | | | | | | | | | | | | | | |
| --- | --- | --- | --- | --- | --- | --- | --- | --- | --- | --- | --- | --- | --- | --- | --- | --- | --- | --- | --- | --- | --- |
| **Plateau of 150ml·min^−1^ in final 60 seconds** | N | Y | Y | Y | Y | Y | Y | Y | Y | Y | Y | Y | Y | Y | N | Y | Y | N | Y | Y | N |
| **%predicted HRmax** | 99% | 104% | 96% | 98% | 94% | 103% | 104% | 94% | 103% | 106% | 93% | 0% | 106% | 109% | 102% | 96% | 96% | 109% | 113% | 99% | 97% |
| **RER** | 1.14 | 1.12 | 1.04 | 1.17 | 1.14 | 1.23 | 1.17 | 1.29 | 1.10 | 1.11 | 1.19 | 1.02 | 1.16 | 1.07 | 0.99 | 1.16 | 1.14 | 1.18 | 1.09 | 1.14 | 1.11 |
| **BLa** | 7.27 | 3.75 | 9.25 | 7.8 | 5.01 | 5.06 | 8.9 | 8.98 | 4.95 | 7.29 | 9.68 | 2.66 | 4.36 | 8.49 | 9.71 | 5.09 | 7.75 | 4.59 | 9.25 | 8.33 | 8.99 |
| **RPE** | 19 | 18 | 19 | 17 | 20 | 19 | 18 | 17 | 18 | 18 | 18 | 19 | 19 | 16 | 15 | 17 | 19 | 19 | 18 | 19 | 19 |
| **Within 10% of predicted HRmax** | Y | Y | Y | Y | Y | Y | Y | Y | Y | Y | Y | N | Y | Y | Y | Y | Y | Y | Y | Y | Y |
| **RER of 1.1** | Y | Y | Y | Y | Y | Y | Y | Y | Y | Y | Y | Y | Y | Y | N | Y | Y | Y | Y | Y | Y |
| **BLa of 8 mmol·L^−1^** | N | N | Y | N | N | N | Y | Y | N | N | Y | N | N | Y | Y | N | N | N | Y | Y | Y |
| **RPE of 19** | Y | N | Y | N | Y | Y | N | N | N | N | N | Y | Y | N | N | N | Y | Y | N | Y | Y |

BLa blood lactate HR heart rate, RER respiratory exchange ratio, RPE rating of perceived exertion
